# Proximal Hypospadias

**DOI:** 10.1100/tsw.2011.76

**Published:** 2011-04-19

**Authors:** Kate H. Kraft, Aseem R. Shukla, Douglas A. Canning

**Affiliations:** ^1^ Division of Urology, The Children's Hospital of Philadelphia, Philadelphia, PA, USA; ^2^ Department of Urologic Surgery, University of Minnesota Amplatz Children's Hospital, Minneapolis, MN, USA; ^3^ Division of Urology, Department of Surgery, University of Pennsylvania School of Medicine, Philadelphia, PA, USA

**Keywords:** buccal mucosa, chordee, penile curvature, proximal hypospadias, urethral meatus, urethroplasty, urogenital folds

## Abstract

Hypospadias results from abnormal development of the penis that leaves the urethral meatus proximal to its normal glanular position. Meatal position may be located anywhere along the penile shaft, but more severe forms of hypospadias may have a urethral meatus located at the scrotum or perineum. The spectrum of abnormalities may also include ventral curvature of the penis, a dorsally redundant prepuce, and atrophic corpus spongiosum. Due to the severity of these abnormalities, proximal hypospadias often requires more extensive reconstruction in order to achieve an anatomically and functionally successful result. We review the spectrum of proximal hypospadias etiology, presentation, correction, and possible associated complications.

## INTRODUCTION

Hypospadias results from abnormal development of the penis that leaves the urethral meatus proximal to its normal glanular position anywhere along the penile shaft, scrotum, or perineum (Fig. 1). A spectrum of abnormalities, including ventral curvature of the penis (chordee), a "hooded" incomplete prepuce, and an abortive corpora spongiosum, are commonly associated with hypospadias.

*Hypospadiology* is a term coined by John W. Duckett, Jr., the former chief of the Division of Urology at the Children's Hospital of Philadelphia (CHOP) and a pioneer in hypospadias repairs. Hypospadiology encompasses a continuously evolving and expanding discipline. While modern experiments have only recently begun to yield a deeper understanding of the genetic, hormonal, and environmental basis of hypospadias, the quest for a surgical procedure that consistently results in a straight penis with a normally placed glanular meatus has occupied surgeons for over 2 centuries. Advances in the understanding of the etiology of hypospadias and the current approaches for the correction of proximal hypospadias to provide a cosmetically and functionally satisfactory repair are the focus of this paper.

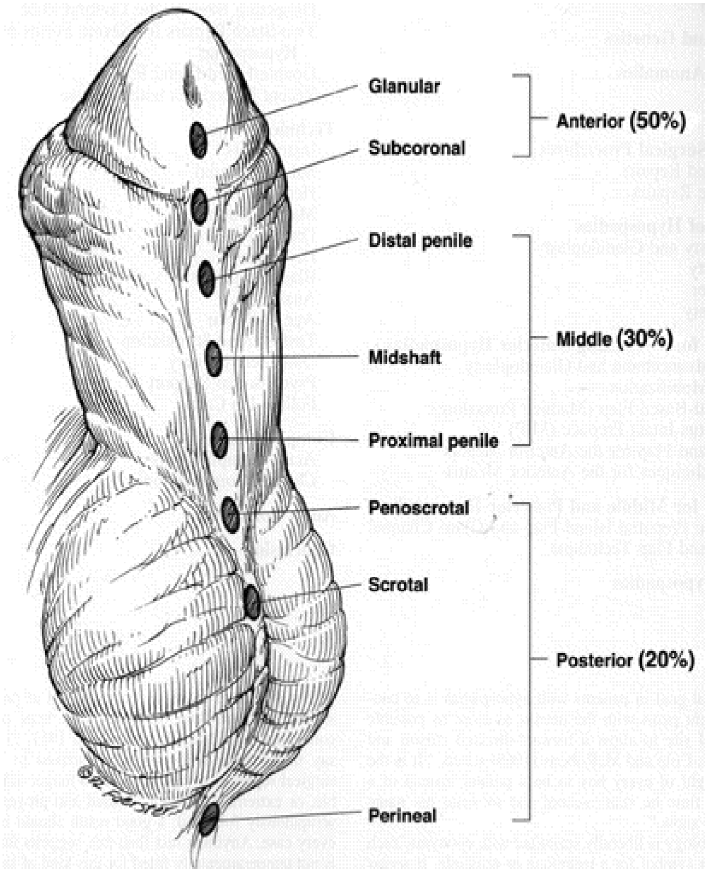

**FIGURE 1.** Classification of hypospadias based on anatomic location of the urethral meatus. Anterior, or distal, hypospadias is the most commonly encountered variant. (From Kraft, K.H., Shukla, A.R., and Canning, D.A. [2010] Hypospadias. *Urol. Clin. North Am.* **37(2),** 167–181. With permission.)

## EPIDEMIOLOGY

The incidence of hypospadias is rising and varies geographically. Prevalence ranges from 0.26/1000 births (both male and female births) in Mexico to 2.11 in Hungary and 2.6 in Scandinavia[1]. A recent study found the rate of hypospadias in a 2-year prospective study to be 38/10,000 live births in the Netherlands, a number six times higher than previously recorded[2]. Sweet and colleagues reported a much lower incidence in Sweden of 1/1250 live male births[3].

In 1997, two independent surveillance systems in the U.S., the nationwide Birth Defects Monitoring Program (BDMP) and the Metropolitan Atlanta Congenital Defects Program (MACDP), reported a nearly doubling of the rate of hypospadias when compared with immediately preceding decades[4]. The incidence of all types of hypospadias increased from 20.2 to 39.7/10,000 live male births during the period from 1970 to 1993, i.e., 1 in every 250 live male births was a boy with hypospadias (measured by BDMP). The MACDP reported a rise in the rate of severe hypospadias of between three- and fivefold. These rising trends, however, may simply reflect earlier diagnosis or an increase in reporting to registries of congenital defects. The increased reporting of more proximal than distal hypospadias cases refutes the argument that these findings simply represent more frequent reporting of minor cases[5].

Recent studies have linked the rising rate of hypospadias in boys born prematurely and small for gestational age, boys with low birth weight, and boys born to mothers over 35 years of age[6,7,8].

Roberts and Lloyd noted an 8.5-fold increase in hypospadias in one of monozygotic male twins compared with singleton live male births[9]. This may suggest a discrepancy in the supply of hCG to the fetus, where a single placenta is unable to meet the requirements of two developing male fetuses.

## ASSOCIATED FINDINGS

### Cryptorchidism and Inguinal Hernia

Between 8 and 10% of boys with hypospadias have a cryptorchid testicle, and 9 to 15% have an associated inguinal hernia[3,10,11]. In boys with more proximal hypospadias, cryptorchidism may occur as frequently as 32%[12]. This strong association between proximal hypospadias and undescended testes further suggests that this clinical entity may represent one end of a spectrum of endocrinopathy. The incidence of chromosomal anomaly in these groups of patients is much higher (22%) than hypospadias (5–7%) or cryptorchidism (3–6%) occurring alone[13,14]. In a series of more than 600 cases of hypospadias, we found that children with associated cryptorchidism and mid-shaft to distal hypospadias had a much higher complication rate when corrected. We are not sure why this occurs, but it may be that a change in the endocrine milieu with the associated cryptorchidism may make the tissues less amenable to correction[15].

### Disorders of Sex Development

Hypospadias and disorders of sex development (DSD) may represent two ends of a spectrum. The more proximal the hypospadias, the more likely a DSD state exists[16]. Rajfer and Walsh reported DSD in 27.3% of boys with a normal-sized phallus, cryptorchidism, and hypospadias[17]. The presence of severe hypospadias and nonpalpable testes with an otherwise normal-looking phallus requires that the urologist test for the presence of a DSD state[16].

### Prostatic Utricle

The prostatic utricle is an elementary structure developing from Mullerian ducts cranially, and from the Wollfian ducts and the urogenital sinus caudally[18]. Boys with hypospadias often have enlargement of the prostatic utricle with resultant urinary tract infections, stone formation, pseudoincontinence and, often, difficult catheterization[19,20,21]. Devine et al. reported that 57% of the patients with perineal hypospadias and 10% with penoscrotal hypospadias had prostatic utricle enlargement demonstrated on urethroscopy. The overall incidence of utricle enlargement in patients with hypospadias was 14% in this series of 44 patients. Utricular enlargement in itself does not indicate DSD, but is seen with increased frequency in patients with 46,XY DSD[22].

## PRESENTATION

The abnormal dorsal prepuce and ventral glans tilt of the newborn penis usually signifies the presence of hypospadias. Further examination of the penis typically reveals the proximally displaced urethral orifice that is often stenotic in appearance, but rarely obstructive. An exception is the megameatus variant of hypospadias. In this unusual case (6% of all distal hypospadias presentations), an intact prepuce is present. The diagnosis is usually not made until after a routine neonatal circumcision is completed[23,24].

The anatomical location of the meatus and extent of ventral curvature, or chordee, should be determined. In some instances, multiple pinpoint dimples may be present on the surface of the urethral plate in addition to the hypospadiac urethral meatus. The meatus is always the most proximal of these defects. Meatal position may be classified as anterior (distal), middle, and posterior (proximal), with more anatomically specific subgroups being further applied (Fig. 2). The meatus is located on the glans or distal shaft of the penis in approximately 70–80% of all boys with hypospadias. Twenty to thirty percent of boys with hypospadias have the meatus located in the middle of the shaft of the penis. The remainder of boys with hypospadias have more severe defects, with the urethral meatus located in the scrotum or even more proximally on the perineum[23,24].

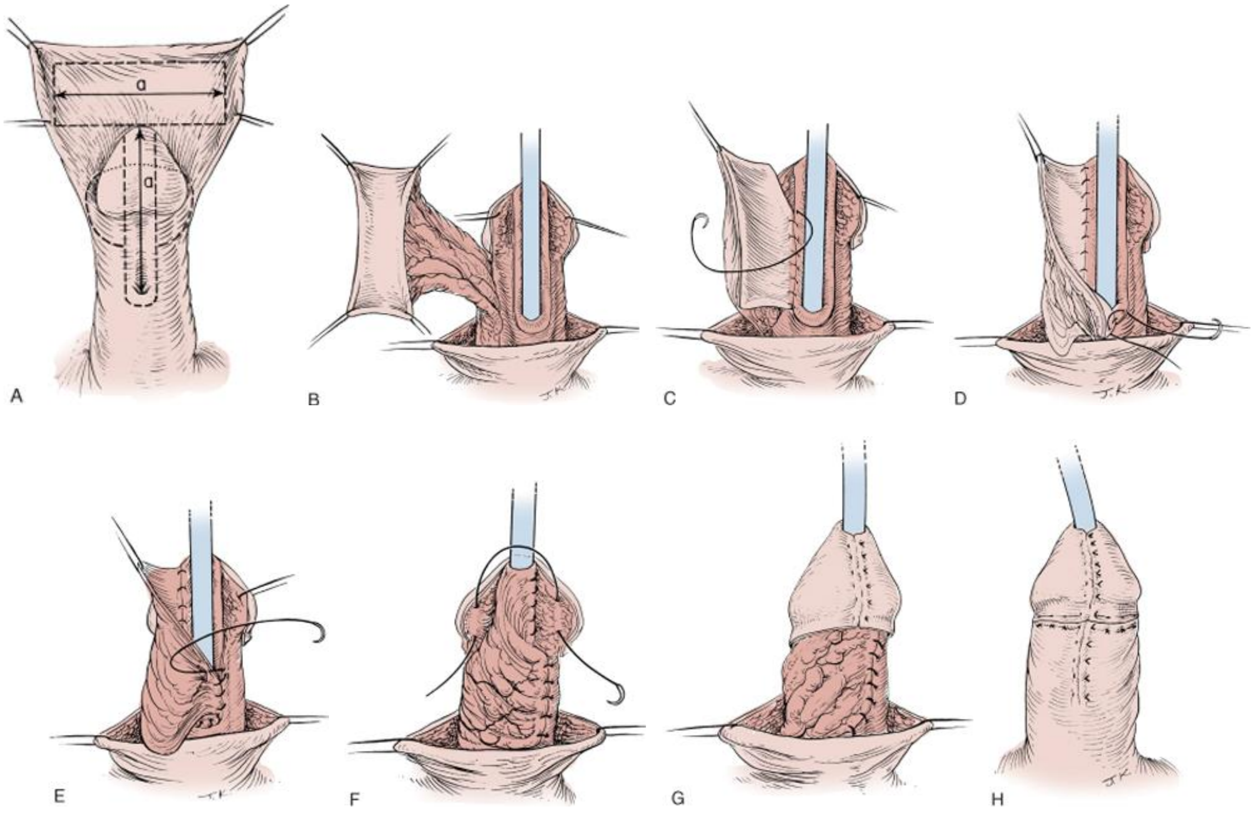

**FIGURE 2.** Onlay island flap repair. (A) Proposed incisions for urethral plate and preputial skin onlay. (B) Pedicled preputial skin onlay with stay sutures. (C) Initial full-thickness suture approximation of onlay flap and urethral plate. (D) Approximation at proximal extent. (E) Completion of anastomosis with running subcuticular technique. (F) Inferolateral border of onlay pedicle has been advanced as a second layer coverage of proximal and longitudinal suture lines. (G) Approximated glans. (H) Completed repair. (From Kraft, K.H., Shukla, A.R., and Canning, D.A. [2010] Hypospadias. *Urol. Clin. North Am*. **37(2),** 167–181. With permission.)

Increased understanding of the endocrinologic origins of hypospadias has corroborated the clinical association of hypospadias with DSD states[17,25]. As such, boys with severe proximal hypospadias and those with hypospadias and cryptorchidism should undergo karyotype analysis and a DSD evaluation as indicated. The uni- or bilaterality of cryptorchidism concomitant with hypospadias does not predict the diagnosis of a DSD state.

A complete penile exam requires independent evaluation of penile length. If the stretched penile length is significantly below the third percentile for age, or if inadequate phallic size precludes surgical repair of hypospadias, then androgen stimulation as pretreatment should be considered. Androgenic pretreatment with hCG has been shown to increase penile length and may also move the meatus to a relatively more distal position as the shaft elongates in response to the hCG[26].

## SURGICAL REPAIR

The goal of hypospadias surgery is a functional sexual organ that is free of curvature. Equally important is a glanular urethral meatus that allows a boy to void with a laminar flow while standing. A cosmetically sound penis requires a cone-shaped glans and supple penile shaft skin.

### Timing of Surgery

Historically, the American Academy of Pediatrics reviews that the ideal age for genital surgery is between 6 and 12 months of age[27]. This age range appears to insulate most children from the psychological, physiological, and anesthetic trauma associated with hypospadias surgery. We prefer, however, to perform the repair at the age of 4 months in infant boys with an adequately sized phallus and without medical problems. Surgery even earlier may be effective in boys with adequate glans volume. Healing seems to occur quickly, with less intense scarring, and young infants overcome the stress of surgery more easily.

## MIDDLE AND PROXIMAL HYPOSPADIAS

We are getting more and more aggressive with tubularized incised plate (TIP) repair for more proximal hypospadias, but we still use the island onlay repair in cases where we are concerned about the width of the urethral plate. If significant curvature exists, we continue to use flap-based repairs.

### Island Onlay Hypospadias Repair

Van Hook first introduced the concept of a preputial flap based on a vascular pedicle to repair proximal hypospadias in 1896[28]. Asopa and colleagues developed the effective use of inner preputial skin for a substitution urethroplasty and Duckett furthered this technique by describing a transverse preputial island flap (TPIF) repair in 1980[29,30]. The island onlay flap evolved from the TPIF as experience demonstrated that repair of the chordee with hypospadias can be accomplished by dissection of the subcutaneous tissue and dorsal midline plication, and that division of the urethral plate is required in only 10% of cases[31]. The concept that spongiosum consists of vascularized tissue and smooth muscle bundles that may be utilized in a hypospadias repair evolved over the 1980s after histologic examination[32,33]. In the past, the onlay island flap was used for more than 90% of our patients with subcoronal hypospadias. We are now using it less in favor of the TIP repair, but it still remains an important part of our hypospadias portfolio.

The circumferential incision begins dorsally 6–8 mm proximal to the corona and is carried ventrally just proximal to the meatus (Fig. 2A). The incision is then carried further proximally to split ventral shaft skin in the midline to the penoscrotal junction. Parallel incisions 5 mm wide or narrower are then made along the urethral plate distally to the glans tip at a point where the flat ventral surface of the glans begins to curve around the meatal groove. We take care to keep these incisions superficial to avoid injury to underlying spongiosum. The skin and dartos fascia are dropped back as residual chordee is released. Dissection of the skin should avoid entering a plane into the intrinsic vascularity of the skin to preserve its viability as a preputial flap. As is commonly the case, if dissection of the penile ventrum reveals thinned spongiosal tissue that is nearly transparent, we incise the urethra proximally to what appears to be normal spongiosum. The urethral plate need not be more than 2 mm wide prior to the onlay transfer.

The island onlay flap is outlined on the inner preputial skin surface with interrupted 5-0 polypropylene sutures that are also used as stay sutures (Fig. 2B). The sutures are grasped so that the fold of tissue between the inner and outer prepuce is accentuated. An 8- to 10-mm segment of this epithelium is sharply divided, with the initial incision just beneath the skin at the junction between the inner and outer preputial faces of foreskin. The combined width of the preserved plate and the flap should be about 10 mm and no wider at the anastomosis than at the urethral meatus.

The freeing of the vascular pedicle begins at the mid-shaft where it is most easily separated from the blood supply to the dorsal penile shaft skin. This approach to the harvest of the flap easily identifies the proper plane and assures preservation of blood supply to the flap (Fig. 3D). The splitting of ventral foreskin completed during the initial circumcising skin incision, in our experience, releases the base of the dorsal vascular pedicle and allows for wider mobilization of preputial foreskin for flap isolation. The flap is then rotated ventrally, or more commonly, transferred by creating a window in the vascular mesentery through which the glans is passed, and then tapered proximally and distally (Fig. 2C). Experience has shown that too wide of a neourethra may lead to kinking or diverticulum formation. The appropriately designed flap is then sutured into place using a lubricated, interrupted 7-0 polyglactic suture at the proximal meatus and then in an interrupted subepithelial fashion along the lateral edges of the plate (Fig. 2D,E). We no longer close the flap over a feeding tube. We prefer to place the tube at the conclusion of the construction of the neourethra. The 8 Fr feeding tube then serves as a spacer to ensure an adequately sized glansplasty. The glansplasty is completed by medial rotation of mobilized glans wings with 6-0 Maxon sutures placed parallel to the cut edge of the glans wing beginning at the urethral meatus (Fig. 2F,G). A 6 Fr Kendall urethral stent is placed and the dorsal preputial skin is split in the midline and rotated ventrally to afford adequate circumferential skin coverage (Fig. 2H).

## Transverse Island Tube Repair

The transverse island tube repair remains a preferred option at CHOP for proximal hypospadias cases that remain amenable to a one-stage repair even after division of the urethral plate to release persistent, severe penile curvature. This procedure incorporates inner preputial skin, as in the onlay technique, to be rolled completely into a neourethra without use of the urethral plate as a vascularized template. A bulky glansplasty, penile torque, and an “oval” rather than “slit-like” meatal result have, however, hampered popular use of the island tube. Incorporating excessive preputial skin into a neourethra has also raised concern for forming a urethral diverticulum and turbulent flow. We describe our recent modifications to this classic procedure that have addressed these cosmetic and functional problems.

Skin incisions facilitate penile degloving to the penopubic junction dorsally and into the penoscrotal junction ventrally. The urethra is opened proximally to healthy vascularized spongiosum as in the onlay repair (Fig. 3A). The urethral plate is then transected at the corona and dissected off of the corporal tissue (Fig. 3B). An artificial erection delineates the extent of residual penile curvature and a Heineke-Mikulicz incision made vertically and closed horizontally straightens the penis (Fig. 3C).

A segment of inner preputial tissue is harvested dorsally as described for the island onlay (Fig. 3D). The pedicled flap is buttonholed and then ventrally transposed. Rather than rolling the tissue into a tube at this point, as previously described, we first anchor the medial margin of the flap to the urethra proximally. This maneuver allows the flap to be optimally tailored by stretching the skin to the opposite, anchored edge of the flap (Fig. 3E). The tube can be fashioned to properly align the anastamosis to the native urethra and to construct a tube of ideal caliber. A second interrupted suture line then rolls the tube effectively into the glans (Fig. 3F).

A glansplasty is completed with 6-0 Maxon sutures placed parallel to the cut edge of the glans as a horizontal mattress to cover the distal edge of the tube. A 6 Fr urethral stent is placed and dorsal preputial skin is fashioned to provide adequate skin coverage as in all hypospadias repairs (Fig. 3G).

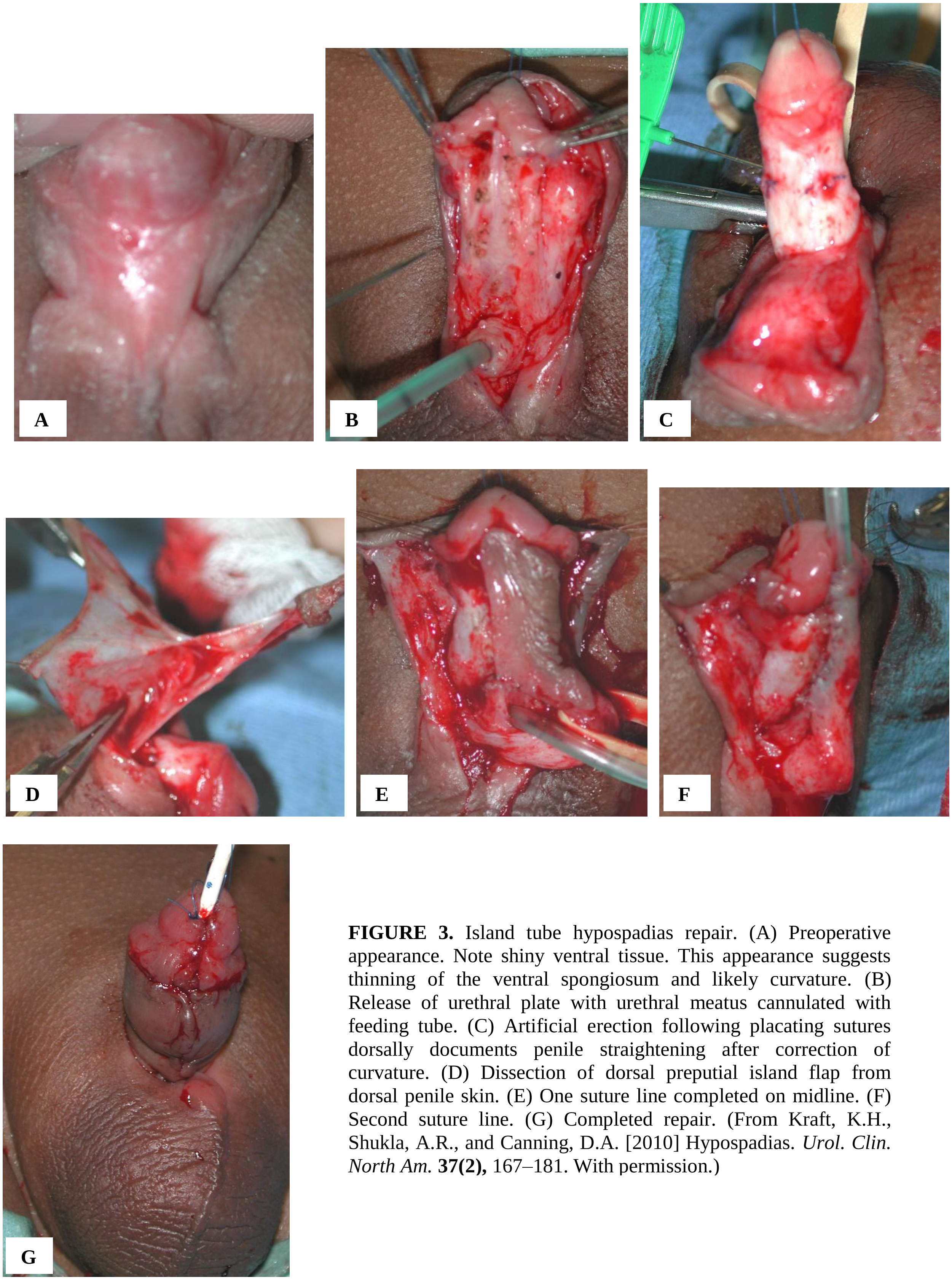

**FIGURE 3.** Island tube hypospadias repair. (A) Preoperative appearance. Note shiny ventral tissue. This appearance suggests thinning of the ventral spongiosum and likely curvature. (B) Release of urethral plate with urethral meatus cannulated with feeding tube. (C) Artificial erection following placating sutures dorsally documents penile straightening after correction of curvature. (D) Dissection of dorsal preputial island flap from dorsal penile skin. (E) One suture line completed on midline. (F) Second suture line. (G) Completed repair. (From Kraft, K.H., Shukla, A.R., and Canning, D.A. [2010] Hypospadias. *Urol. Clin. North Am.* **37(2),** 167–181. With permission.)

## Two-Stage Repair

We occasionally encounter challenging cases where severe chordee and a proximal meatus limit the applicability of a one-stage tube repair. In a few cases, injury to the vascular pedicle of the only tube during harvest requires that the repair be staged. Anecdotal and reported experience maintains, in fact, that the two-stage technique offers fewer complications overall and better cosmetic results than the single-stage repair for select cases[34,35]. In cases where curvature is a great challenge and we are concerned that we may need to monitor the success of the repair, we have elected to stage the repair[36,37]. In our experience, however, we have found a similar fistula rate following the second stage of a two-stage repair to that following a one-stage island onlay or tube repair[38].

A two-stage repair often involves a scrotoplasty with an aggressive attempt to relieve penile curvature, including transection and proximal removal of the plate. Either a dermal graft or tunica vaginalis may be interpositioned, although very rarely in our experience, to bridge any defect in the ventral tunica albuginea surface. Preputial skin at the dorsum is then split to rotate the resultant flaps ventrally. These flaps are allowed to settle into place and represent the future urethral plate (Fig. 4A–F).

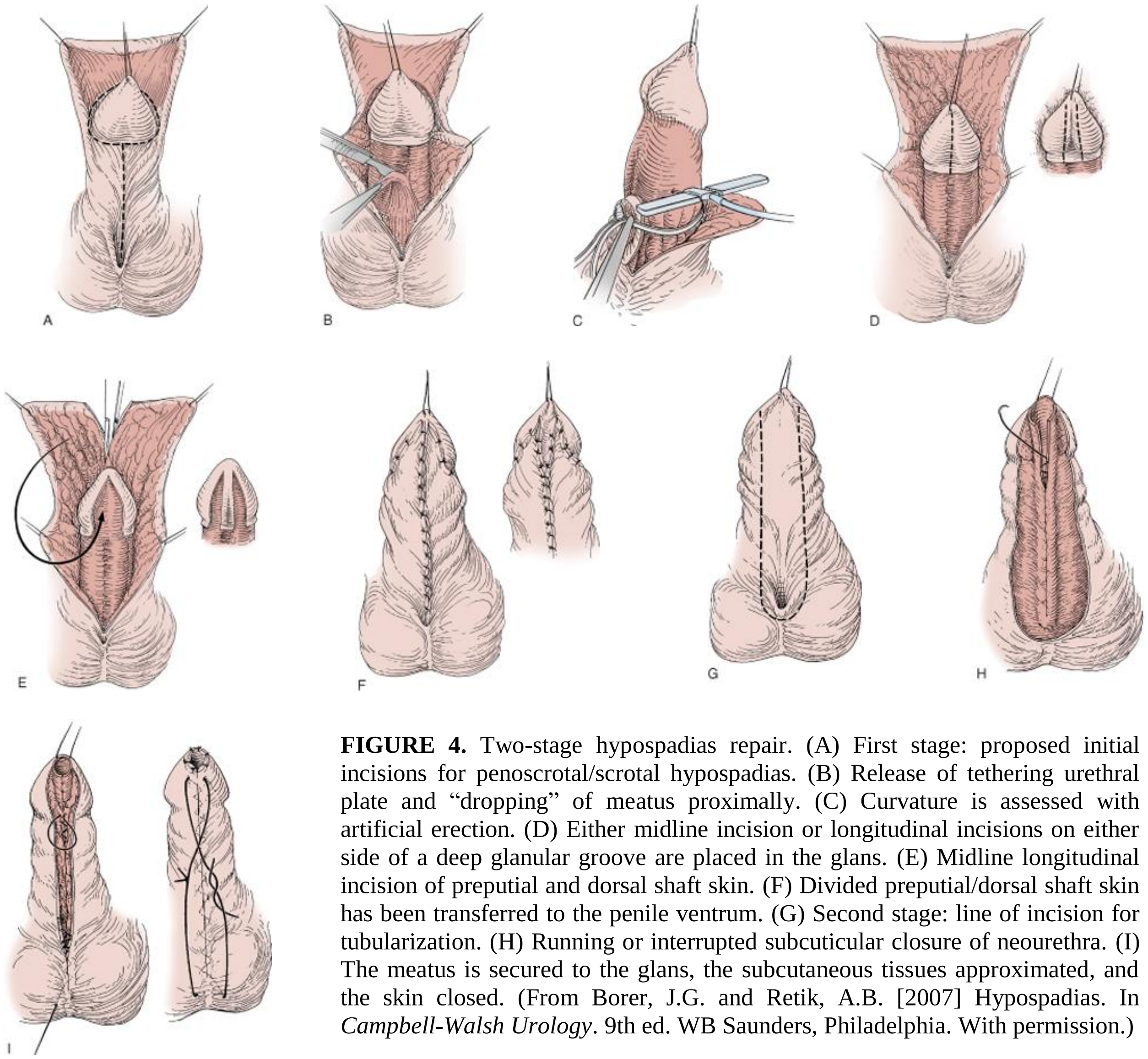

**FIGURE 4.** Two-stage hypospadias repair. (A) First stage: proposed initial incisions for penoscrotal/scrotal hypospadias. (B) Release of tethering urethral plate and “dropping” of meatus proximally. (C) Curvature is assessed with artificial erection. (D) Either midline incision or longitudinal incisions on either side of a deep glanular groove are placed in the glans. (E) Midline longitudinal incision of preputial and dorsal shaft skin. (F) Divided preputial/dorsal shaft skin has been transferred to the penile ventrum. (G) Second stage: line of incision for tubularization. (H) Running or interrupted subcuticular closure of neourethra. (I) The meatus is secured to the glans, the subcutaneous tissues approximated, and the skin closed. (From Borer, J.G. and Retik, A.B. [2007] Hypospadias. In *Campbell-Walsh Urology*. 9th ed. WB Saunders, Philadelphia. With permission.)

The second stage is planned after an interval of about 6 months. At that point, parallel vertical incisions 12–15 mm apart are mapped distally, beginning at the meatus and including the glans. Glans wings are mobilized as in the Thiersch-Duplay repair and, if also needed, the glans may be incised in the midline as with the TIP repair. Incisions are completed and a neourethra is then tubularized to complete the repair using a standard Thiersch-Duplay technique (Fig. 4G–I). We aggressively cover the repair with dartos tissue or, in some cases, with processus vaginalis flaps taken adjacent to the spermatic cord on one side or the other. In some cases, placement of the suture line into the scrotum facilitates healing (Fig. 4I). The penis is then mobilized from the scrotal flaps in a third stage, usually 3–12 months later.

## Free Grafts

Severe proximal hypospadias and repeat hypospadias repairs may incorporate free grafts to construct a neourethra or augment an existing plate. The versatility of most primary hypospadias repairs using preputial skin or the urethral plate, however, has obviated our use of free grafts for primary hypospadias repairs. In the past, at our institution, we reserve this technique only for reoperations or the rare instances where a paucity of local tissue was evident[39]. Free skin, bladder mucosa, tunica vaginalis, and buccal mucosa have variously been described as appropriate tissues for free graft use[40,41,42].

Since its description by Bracka in 1995, the staged buccal mucosa graft has become increasingly accepted in hypospadias surgery[43]. In addition to cases of repeat repair with significant scarring, the Bracka approach may be indicated in severe proximal hypospadias cases. The procedure requires removal of all urethral plate or scar tissue, and substitution with a buccal mucosa graft sutured to the corpora cavernosa. The mucosa is then tubularized 6 months later once the graft take is stable. Advantages of this technique include a well-vascularized graft for urethral reconstruction and minimal associated complications[43,44]. The buccal mucosa is an excellent substitute for the urethra as it is designed to remain moist and has no potential for hair growth[43].

Our experience has been that the bladder mucosa is less pliable than buccal mucosa, with the latter being less likely to shrink and requiring a one-to-one ratio for harvest compared to the defect to be repaired. Additionally, the thick epithelium, tensile strength, and high levels of type IV collagen found in buccal mucosa favor graft take[45]. Use of bladder mucosa has been abandoned in favor of buccal mucosa in the rare cases requiring a free graft.

We prefer to harvest buccal mucosa from the lower lip, although the inner cheek may be used with care to avoid Stensen’s duct (Fig. 5A). After graft harvest, the lip donor site may be left to heal by secondary intention to avoid contracture and distortion. Cheek donor sites can be closed primarily with absorbable sutures provided the defect is not significantly large. The graft is placed on a scaffold to tailor it for onlay. Adipose tissue is excised to expose the subepithelium and multiple perforations are made in the graft with an 18-gauge needle. This creates a surface that optimizes graft take and allows evacuation of accumulated fluid or blood, thereby preventing hematoma formation and subsequent disruption. The graft is trimmed to size if used as an onlay or tailored to be wrapped into a neourethra, without allowing for contracture in the case of buccal mucosa (Fig. 5B). The edges of the graft epithelium are sutured to the surrounding shaft skin, and several plicating sutures are placed to approximate the graft directly to the base and facilitate neovascularization. We stent repairs completed with free grafts for 10–21 days depending on location and length. First-stage repairs with grafts are dressed with glycerine-soaked cotton secured with nonabsorbable sutures, followed by a sandwich dressing. In most cases, we place a suprapubic catheter into the bladder under cystoscopic guidance to divert the urinary stream from the fresh graft.

Metro et al. and Hensle et al. have reported long-term results for buccal mucosa grafts in complex hypospadias reoperations[39,46]. Overall complication rates between 32 and 57% were reported, with graft stricture and meatal stenosis being most common. All complications were evident by 11 months in both groups and were seen less commonly as the surgical experience widened. The buccal mucosa remains a viable nongenital tissue alternative for a select subgroup of patients requiring urethral reconstruction.

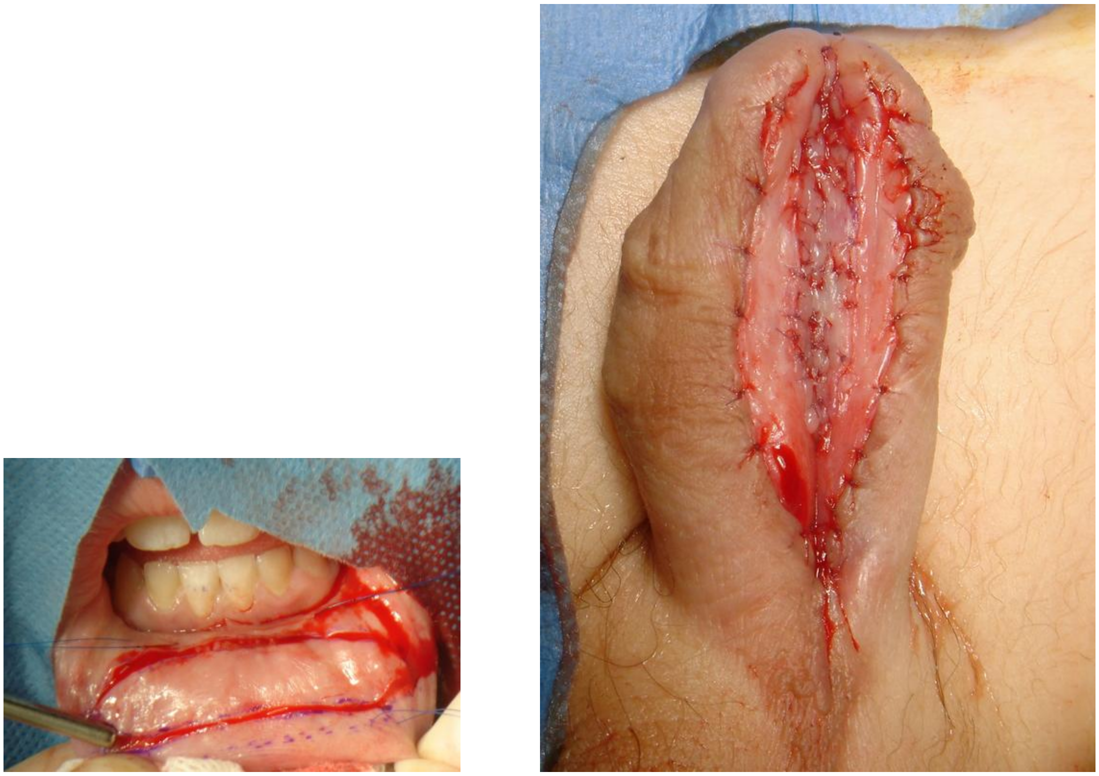

**A** **B**

**FIGURE 5.** Staged buccal mucosa repair for failed hypospadias. (A) Lip harvest site for graft. (B) Graft sutured into place along midline of strictured urethral plate. (From Kraft, K.H., Shukla, A.R., and Canning, D.A. [2010] Hypospadias. *Urol. Clin. North Am.* **37(2),** 167–181. With permission.)

## COMPLICATIONS

### Urethrocutaneous Fistula

The postoperative appearance of a urethrocutaneous fistula remains one of the most frustrating, although increasingly rare, complications of hypospadias surgery today. Adaptations in technique, including strict adherence to the principles of plastic surgery in tissue handling, avoiding suture line overlap, and the transposition of additional tissue layers, have cumulatively contributed to minimizing the incidence of failure. The team at CHOP has previously reported a <5% fistula rate for the island onlay hypospadias repair[47].

When a fistula is diagnosed, whether as a perioperative or delayed complication, at least 6 months (and more if an older boy) should be allowed to elapse prior to repeat surgical intervention. This interval will allow inflammation and edema to resolve, and enable an accurate assessment of the viability and suitability of local tissue to be incorporated during reoperation. While a small proximal fistula may be approached by excising the fistula tract to the urethra and performing an inverting closure with additional adjacent tissue, a larger fistula or multiple small fistulae often require a preputial or dartos-based skin flap for an onlay closure. Transposition of considerable dorsal tissue to the ventrum with an originally completed onlay repair will provide enough tissue for a primary closure at reoperation. We have found that the smaller, distal fistulae represent a deceptively more complex problem, as repair often requires an onlay to ensure adequate cover as well as a reconstruction of the distal glansplasty.

In the complex hypospadias patient with urethrocutaneous fistula or severe stricture disease, excision of all abnormal tissue with onlay of buccal mucosa in a two-stage repair has proven successful in our experience. Hosseini and colleagues describe a 78.6% overall success rate with use of buccal mucosa grafts in patients who developed fistulae following previous hypospadias repair[48]. Evaluation of staged repairs with buccal mucosa grafts in hypospadias cripples suggests that this approach provides good results[49].

Use of buccal mucosa grafts in the complex hypospadias patient can result in complications, however, with rates of meatal stenosis as high as 12.5%, urethral stricture in 31%, and urethrocutaneous fistula in up to 25%[50]. These data are based on small patient cohorts with short follow-up intervals, however, and further investigation is mandated.

## Urethral Diverticula and Meatal Stenosis

Urethral diverticula may occur as an independent complication or as a secondary consequence of meatal stenosis. Incorporating excessive tissue in a primary closure or incomplete tailoring of an onlay flap increases the likelihood of diverticula formation. The diverticulum may be trimmed, followed by closure and the overlay of local tissue. We are cautious about overzealous narrowing of the diverticulum. The tissue that makes up the diverticulum is less distensible laterally. Aggressive narrowing of the urethroplasty, particularly if the defect is long, may result in higher voiding pressures and flattened micturition curves. We prefer to wait up to 1 year to repair diverticula following an onlay or tube repair, as local revascularization of the flap in that interval allows for transection of the pedicle if required at the time of reoperation.

Urethral meatal stenosis occurs if blood supply to the distal urethra is compromised following hypospadias repair. Several technical modifications have sought to limit its occurrence. Limiting the involvement of the very distal urethral plate during a TIP or MAGPI (meatal advancement and glanduloplasty) procedure and avoiding an excessively tight glansplasty are now understood to be important measures to avoid meatal stenosis.

## SUMMARY

Hypospadias results from abnormal development of the penis, leaving the urethral meatus proximal to its orthotopic glanular position. The meatus may be located anywhere along the penile shaft and the spectrum of concomitant abnormalities includes ventral curvature of the penis, a dorsally redundant prepuce, and atrophic corpus spongiosum. Due to the severity of these abnormalities, proximal hypospadias often requires more extensive reconstruction to achieve a successful repair. Familiarity with a variety of techniques and adherence to surgical principals, such as maintaining adequate vascular supply and multilayer closure, can achieve both a cosmetically and functionally satisfactory result.
